# Transversal approach via a bladder neck and prostate combined longitudinal incision versus standard approach of robotic-assisted radical prostatectomy for localized prostate cancer: a retrospective analysis

**DOI:** 10.1186/s12885-024-12015-0

**Published:** 2024-03-06

**Authors:** Zefan Shen, CenChao Yao, YuChen Bai, YiFan Wang, Qi Zhang

**Affiliations:** 1 Urology and Nephrology Center, Department of Urology, Zhejiang Provincial People’s Hospital, Affiliated People’s Hospital, Hangzhou Medical College, Hangzhou, Zhejiang 310014 China; 2 Graduate Department, Bengbu Medical College, Bengbu, Anhui 233000 China; 3 The Second Clinical Medical College, Zhejiang Chinese Medical University, Hangzhou, Zhejiang 310000 China

**Keywords:** S-RALP, L-RALP, Prostate cancer, EPIC-CP, Robotic-assist

## Abstract

**Background:**

Transversal approach for robotic-assisted radical prostatectomy via a bladder neck and prostate combined longitudinal incision (L-RALP) is a novel surgical method for patients with respectable prostate cancer.

**Methods:**

There were 669 patients with prostate cancer underwent L-RALP or S-RALP which identified from April 2016 to April 2020. The perioperative outcomes, Expanded Prostate Cancer Index Composite for Clinical Practice (EPIC-CP) scores, sexual function and urinary control ability were included and compared between two groups.

**Results:**

In the 669 patients, 277 of them were included into the final analysis. 151 patients received S-RALP and 126 received L-RALP. Baseline features were balanced. Patients in the S-RALP group had significantly shorter average surgical time (135.93 vs 150.04 min; *p* < 0.001) than those in L-RALP group. Intraoperative bleeding volume, early postoperative complications rates, postoperative catheter removal time and hospital stays were comparable between two groups. There was no difference in biochemical recurrence at 3, 6, 12 and 18 months of follow-up. Of note, the urinary control function recovers of patients in the L-RALP group was significantly better than those in the S-RALP group. Moreover, patients in the L-RALP group had much better results of EPIC-CP (including urinary control and total score) than those in the S-RALP group at 6 week and 3, 6, 12 and 18 months.

**Conclusions:**

Both S-RALP and L-RALP were safe and effective with similar long-term clinical outcomes in patients with respectable prostate cancer. Patients received L-RALP had significantly better postoperative outcomes including urinary control, and recovery period.

**Supplementary Information:**

The online version contains supplementary material available at 10.1186/s12885-024-12015-0.

## Introduction

Prostate cancer is one of the most common malignant tumors in males, with approximately one million new cases diagnosed globally in one year. With the increasing aging of the population, the average age of onset of prostate cancer is also becoming younger than before. Moreover, with the continuous improvement of diagnostic techniques, more and more cases could be diagnosed with early-stage prostate cancer [1]. In 2020, there were nearly 1.4 million new cases and 375,000 deaths worldwide, making prostate cancer the second most common cancer-related cause of death in men [2].

Since RP is only one of the options for localized PCa who have the opportunity for surgery, with significant impacts on male sexual function and urinary control. Urinary incontinence (UI) [3] and erectile dysfunction (ED) [4] are the most common postoperative complications, significantly influencing the patients’ quality of life and lifestyle after treatment. Hence, protecting male sexual function and urinary control is an important issue in this field. How to maximize the preservation of patients’ urinary control and erectile function has become a major concern for patients with localized prostate cancer [5].

In 2000, Binder et al. published the first study on robot-assisted laparoscopic radical prostatectomy (RALP) for patients with localized prostate cancer [6]. Gradually, RALP has been widely performed in clinical practice because of its highly flexible mechanical arms, extremely precise surgical instruments, and clearer 3D surgical vision. Moreover, robot-assisted surgery could avoid damage to the surrounding prostate tissue, reduce intraoperative bleeding, better protect functional nerves, reduce the positive rate of surgical margins, and improve the overall prognosis [7–12]. Although many studies have revealed that the positive margin rate and recurrence rate after robot-assisted laparoscopic prostatectomy (RALP) are significantly lower than those after laparoscopic radical prostatectomy (LRP), the problems of postoperative UI and ED remained unexplored [13, 14].

Recently, our group reported a new surgical technique, named the transversal approach for robotic-assisted radical prostatectomy via a bladder neck and prostate combined longitudinal incision (L-RALP). This method enters through the anterior approach without opening the pelvic fascia. Not only it have a better surgical view under the longitudinal incision, but also it maximizes the preservation of important tissue structures around the prostate, which has unique advantages in preserving postoperative erectile function and urinary control in prostate cancer patients. Here, we summarized the clinical data of 669 patients with prostate cancer who underwent L-RALP or S-RALP from April 2016 to April 2020 in our center and aimed to compare the initial perioperative and postoperative outcomes of patients received L-RALP with those treated with S-RALP.

## Material and method

### Patients’ inclusion

**Fig. 1:**
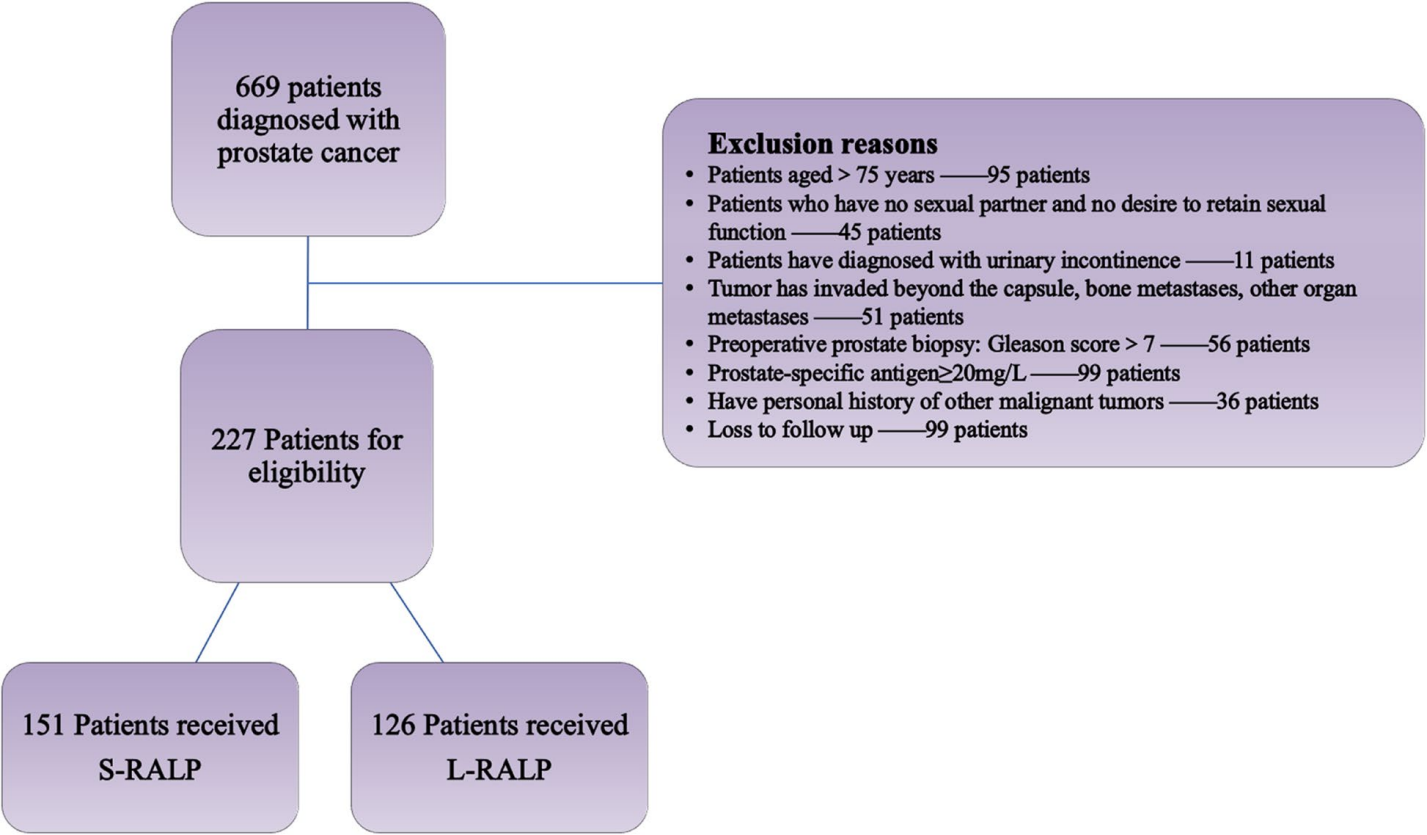
The major exclusion criteria

This study retrospectively identified 669 patients with histologically confirmed prostate cancer who underwent Da Vinci robotic-assisted laparoscopic radical prostatectomy (either S-RALP or L-RALP) in our hospital from April 2016 to April 2020. The major exclusion criteria are shown in Fig. 1. The major clinicopathological characteristics include (i) preoperative: age, height, weight, body mass index (BMI), prostate-specific antigen (PSA), enhanced magnetic resonance imaging (MRI) of the prostate, Prostate Imaging–Reporting and Data System (PIRADS) score, preoperative clinical TNM staging, and preoperative Expanded Prostate Cancer Index Composite for Clinical Practice (EPIC-CP) scores; (ii) perioperative: surgical time, intraoperative bleeding, postoperative hospitalization, postoperative complications, pathologic and oncologic data, Gleason score, prostate volume, pathological TNM staging, lymph node involvement; (iii) postoperative: EPIC-CP scores of patients at 3 weeks, 3 months, 6 months, 12 months, and 18 months after surgery, biochemical recurrence of patients, postoperative recovery of urinary control function, were collected and compared between two groups. All enrolled patients received complete preoperative examinations, including electrocardiography, chest X-ray, enhanced MRI of the prostate, complete blood count, blood biochemistry, tumor markers, and other relevant tests. Two weeks before surgery, antiplatelet and aspirin medications was discontinued, and low-molecular-weight heparin and physical anticoagulation measures were used to prevent thrombosis in these patients during the perioperative period.

All eligible patients must undergo a prostate biopsy before radical prostatectomy, and pathological grading and clinical staging of malignant prostate tumors for each patient would be performed based on the biopsy pathology report and prostate imaging data. Then, a surgical plan will be developed for each patient, and the patient and his family will be informed of the surgical risks and sign the surgical consent form. The study protocol was approved by the ethics committee and institutional review board of our center and conducted according to the Declaration of Helsinki, Guidelines for Good Clinical Practice, and local laws and regulations of China. All patients would receive radical prostatectomy within 3 weeks after the biopsy to avoid difficulties in surgical dissection and blurred vision caused by tissue inflammation and chronic bleeding after the biopsy. Two clinical urologists with the same assistant will perform the surgeries for both groups. The two surgeons have more than 10 years of experience and have performed over 300 RALP surgeries before this study.

### Surgical technique

#### S-RALP

As a modified approach to the standard da Vinci surgical robot-assisted laparoscopic prostatectomy, we have implemented the following surgical principles to control variables [15]: bladder neck preservation, endopelvic fascia preservation, tension-free nerve sparing, and urethral length preservation. Two lead surgeons communicated with each other and followed these surgical principles [16, 17].

#### L-RALP

**Fig. 2:**
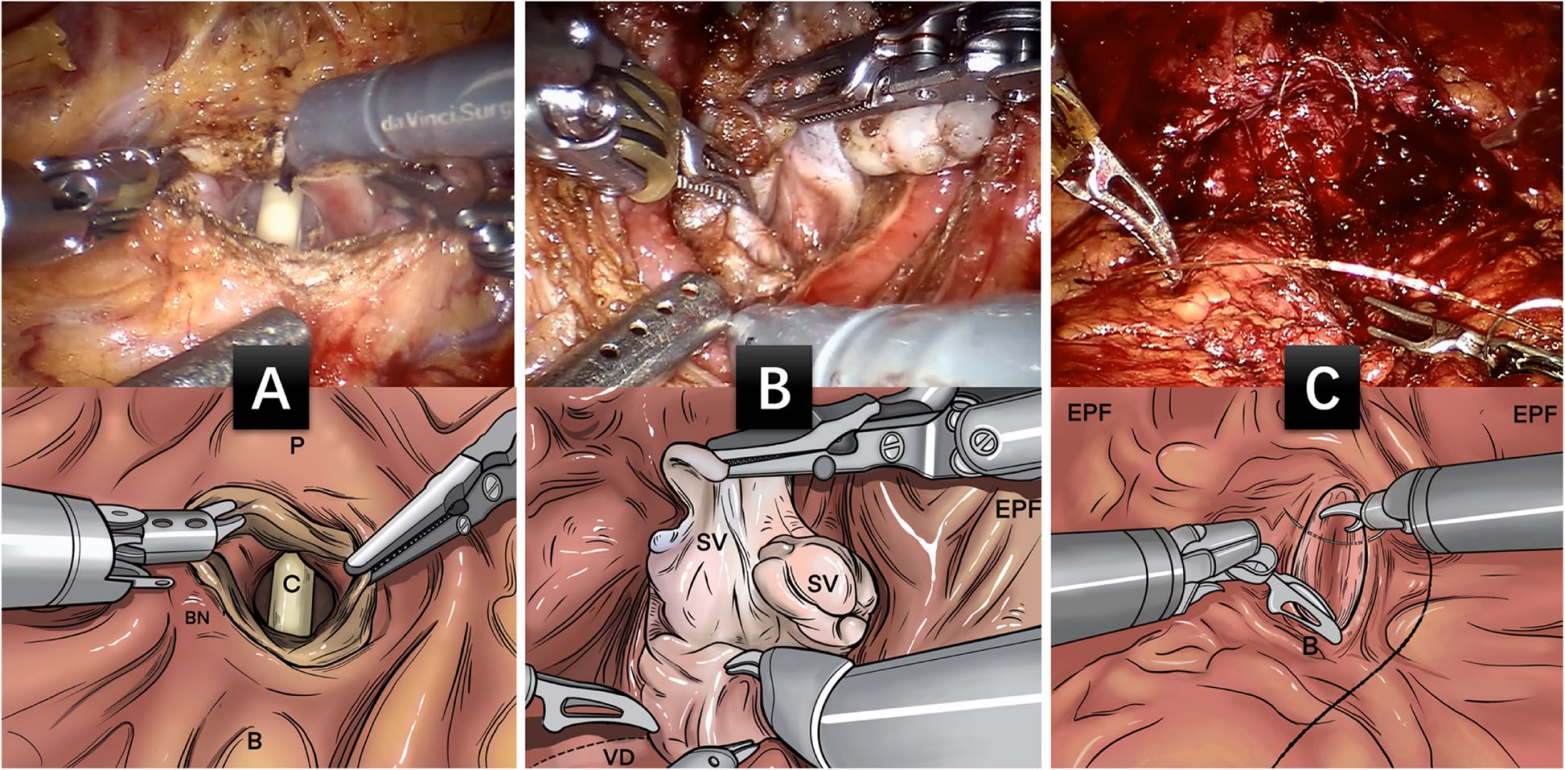
Schematic diagram of the L-RALP. **A** longitudinal bladder incision was made on the bladder neck and prostate; **B** bladder prostatic muscle was incised in order to expose the bilateral vas deferens and seminal vesicles; **C** Anatomical reduction; P: prostate; C: catheter; B: bladder; BN: bladder neck; SV: seminal vesicles; VD: Vas deferens; EPF: endopelvic prostate fascia

The positioning and arm placement method of L-RALP is the same as that of S-RALP. First, space is established around the prostate through the first peritoneal fold on the back of the bladder. The connective tissue of the retroperitoneal space of the bladder is then dissociated into the retropubic space, and the fatty tissue above the prostate is then cauterized to expose the pubic prostate. Secondly, the neck of the bladder was determined by extubating, and a longitudinal bladder incision (3–5 cm) was made between the neck of the bladder and the prostate (Fig. 2A). Open the bladder to expose the neck and make a 360° incision around the neck. Determine the ureteral opening and remove the incision from the ureteral opening. The posterior lip of the bladder neck was opened, and the incision was extended along the posterior margin of the prostatic external capsule to the 5th and 7th points of the bladder neck. The vesicoprostatic muscle was cut open to reveal the bilateral vas deferens and seminal vesicles (Fig. 2B). Third, the prostate tissue was removed from the vas deferens on both sides and the seminal vesicles were isolated. The posterior wall of the prostate in front of the Denon Villiers fascia is opened to expose the prostate sac and enlarge the plane of separation between the rectum and the prostate to the lateral prostatic ligament. In the fourth step, pneumoperitoneum pressure is raised to 18–20 mmHg, then the plane is separated in the inner fascia layer on both sides of the prostate capsule to the apex. Without cutting the puboprostatic ligament, the urethra was exposed by electrotomy along the prostatic envelope to cut off the DVC attachment. Cut the anterior wall of the urethra with scissors, remove the catheter, and then cut the posterior wall of the urethra, taking care to retain sufficient length of urethra tissue. Remove the prostate and check to make sure the prostate sac is intact. If the wound is bleeding, the catheter can be removed to compress the wound to stop bleeding. Fifth, vesicourethral double needle barb anastomosis: starting at 6 o’clock 3–0 double needle single Joe barb anastomosis—reconstruction of the bladder neck. The perineum can be lifted to better expose the urethral stump and reduce the tension of the vesicoureterostomy. After 2–3 stitches were sutured on both sides of the posterior wall, the suture lines were tightened to make the posterior wall of the vesicoureteral anastomosis completely closed. The two sutures were closed clockwise and counterclockwise to close the bladder incision (Fig. 2C). During reconstruction, care should be taken to avoid damaging or stretching the ureteral opening. The anterior bladder tissue closed and was anatomically reduced. The F18 double-cavity catheter was replaced, a pelvic drainage tube was placed, and specimens were collected [18].

### Statistical analysis

SPSS 26.0 software was used for statistical analysis to describe the characteristics of the two groups of cases. Independent sample t-tests were performed on age, BMI, PSA, MRI PIRADs score, surgery time (min), intraoperative blood loss (ml), prostate volume, lymph node metastasis, positive surgical margin rate, postoperative biochemical recurrence, clinical stage, etc. Ordered categorical data such as Gleason score was subjected to multiple group rate chi-square tests. Independent sample t-tests were used to compare the Epic-cp scores of the two groups. Kaplan–Meier analysis was performed on the postoperative urinary control recovery time of the two groups of patients, and the recovery time curve was drawn using R language. All data analysis was conducted with a significance level of *p* < 0.05.

## Results

### Baseline parameters of study population

We initially identified 669 patients with prostate cancer who received RALP and 277 of them were eligible:151 in the S-RALP and 126 in the L-RALP group. Baseline clinical parameters are listed in Table 1. There were no statistically significant differences in age, BMI, PSA, Gleason score, clinical stage, and preoperative baseline EPIC-CP scores between the two groups. The preoperative clinical stage evaluations of both groups were within T2c, and the pathological Gleason scores were less than 7 which is revealed in Table 2.

**Table 1:** Preoperative baseline characteristics of all included patients

	S-RALP(*N* = 151)	L-RALP(*N* = 126)	*P* value
Age (year), mean ± SD	67.00 ± 5.54	66.24 ± 6.22	0.282
BMI (kg/m^2^), mean ± SD	23.77 ± 2.99	23.87 ± 3.13	0.779
PSA (ng/ml), mean ± SD	9.47 ± 3.58	9.63 ± 3.59	0.722
MRI PIRADS score, mean ± SD	4.09 ± 0.52	3.96 ± 0.65	0.061
TNM stage no. (%)			
T2a	18(11.9%)	14(11.1%)	0.873
T2b	94(62.3%)	76(60.3%)	
T2c	39(25.8%)	36(28.6%)	
Gleason score no. (%)			
6	51(33.8%)	49(38.9%)	0.378
7	100(66.2%)	77(61.1%)	
Baseline EPIC-CP urinary incontinence score, mean ± SD	1.19 ± 0.96	1.37 ± 0.94	0.053
Baseline EPIC-CP sexual function score, mean ± SD	2.99 ± 1.26	3.19 ± 1.34	0.111
Baseline EPIC-CP total score, mean ± SD	8.13 ± 4.26	8.19 ± 2.07	0.760

*BMI* Body mass index, *PSA* Prostate-specific antigen, *EPIC-CP* Expanded Prostate Cancer Index Composite for Clinical Practice, *MRI* Magnetic resonance imaging, *PIRADS* Prostate Imaging Reporting and Data System, *L-RARP* Transvesical approach for robotic-assisted radical prostatectomy via a bladder neck and prostate combined longitudinal incision, *S-RARP* Standard robot-assisted radical prostatectomy, *SD* Standard deviation

**Table 2:** Perioperative data of two groups

	S-RALP(*N* = 151)	L-RALP(*N* = 126)	*P* value
Surgical time (min), mean ± SD	135.93 ± 27.23	150.04 ± 26.36	< 0.001
Intraoperative bleeding (mL), mean ± SD	85.70 ± 62.58	85.92 ± 65.03	0.619
Postoperative hospital stay (day), mean ± SD	4.03 ± 1.47	3.79 ± 1.27	0.150
postoperative catheter removal (day), mean ± SD	5.41 ± 0.65	5.45 ± 0.67	0.597
Early postoperative complications, no. (%)	3(1.9%)	1(0.8%)	0.409

*L-RARP* Transvesical approach for robotic-assisted radical prostatectomy via a bladder neck and prostate combined longitudinal incision, *S-RARP* Standard robot-assisted radical prostatectomy, *SD* Standard deviation

### Perioperative data

The average surgical time of the S-RALP group (135.93 ± 27.23 min) was significant shorter than that of the L-RALP group (150.04 ± 26.36 min) (*p* < 0.001). However, there were no statistically significant differences between the S-RALP and L-RALP groups (Table 2) in terms of intraoperative blood loss (85.70 vs. 85.92 mL, *p* = 0.619), time to removal of the urinary catheter after surgery (5.41 vs. 5.45 days, *p* = 0.597), length of hospital stays after surgery (4.03 vs. 3.79 days, *p* = 0.150), and rate of perioperative complications (1.9% vs. 0.8%, *p* = 0.409).

### Pathologic outcomes

Pathologic outcomes of patients from the S-RALP and L-RALP groups were summarized in Table 3. There were no significant differences in prostate volume (22.39 vs. 22.72 mL, *p* = 0.619), pathologic stage (T2: 89.4 vs. 95.2%; T3: 10.6 vs. 4.8%; *p* = 0.893), Gleason score (*p* = 0.378), lymph node involvement (*p* = 0.672) and positive margin (*p* = 0.191).

**Table 3:** Pathological information

	S-RALP(*N* = 151)	L-RALP(*N* = 126)	*P* value
Prostate volume (ml), mean ± SD	22.39 ± 5.19	22.72 ± 6.30	0.628
Pathologic stage, no (%)			
T2	135(89.4%)	120(95.2%)	0.893
T3a or T3b	16(10.6%)	6(4.8%)	
Gleason score, no(%)			
6	44(29.1%)	40(31.8%)	0.378
7	81(53.6%)	71(56.3%)	
8	25(16.6%)	14(11.1%)	
9	1(0.7%)	1(0.8%)	
Lymph node involvement, no(%)	2(1.3%)	1(0.8%)	0.672
Positive margin, no(%)	11(7.3%)	15(11.9%)	0.191

*L-RARP* Transvesical approach for robotic-assisted radical prostatectomy via a bladder neck and prostate combined longitudinal incision, *S-RARP* Standard robot-assisted radical prostatectomy, *SD* Standard deviation

### Postoperative outcomes

**Fig. 3:**
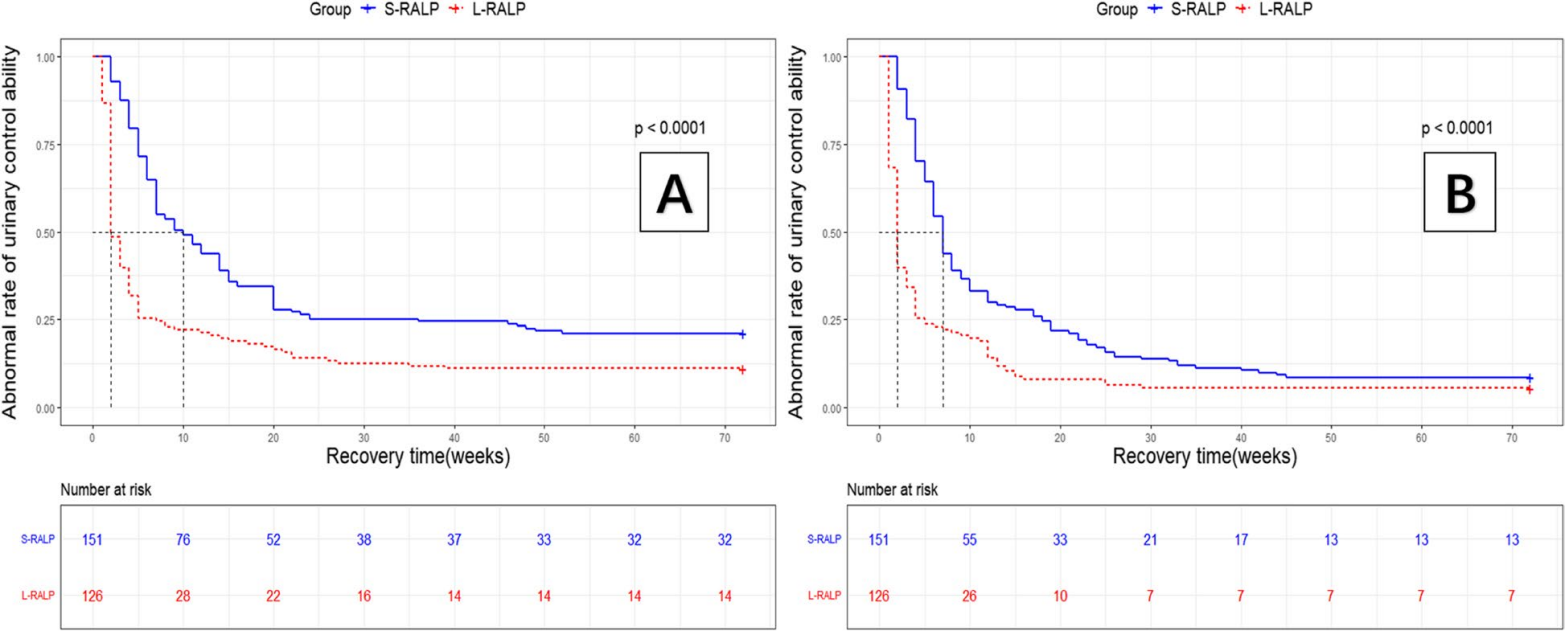
Recovery period of the two groups. Discrimination of two groups was evaluated by the dotted red line and blue line curve which represents L-RALP and S-RALP. The end point of figure (**A**) was set up to 0 pad and the end point of figure (**B**) was set up to 0-1 pad

Overall, there were no significant differences in biochemical recurrence (*p* = 0.348), time to biochemical recurrence (*p* = 0.825), and rates of adjuvant therapy (*p* = 0.932). However, in terms of urinary control function, the recovery of patients in the S-RALP group is far worse than those in the L-RALP group. The L-RALP group has a significantly higher number of patients who can recover to 0 pad per day compared to the S-RALP group (88.9% vs. 78.8%, *p* = 0.025; Table 4). Furthermore, the recovery period of the L-RALP group is also much shorter than that of the S-RALP group (0 pad: 2 vs. 7 weeks, *P* < 0.001; Fig. 3A. 0–1 safety pad: 2 vs. 6.5 weeks, *p* < 0.001; Fig. 3B).

**Table 4:** Postoperative outcomes

	S-RALP(*N* = 151)	L-RALP(*N* = 126)	*P* value
Biochemical recurrence, no. (%)	17(11.2%)	19(15.1%)	0.348
Time to Biochemical recurrence, (w)	24(4–36)w	36(8–60)w	0.825
Adjuvant therapy, no. (%)	33(21.9%)	27(21.4%)	0.932
Continence at follow-up, no (%)			
0-Pad	119(78.8%)	112(88.9%)	0.025
0–1 safety Pad	138(91.4%)	119(94.4%)	0.330
Time to continence (d), median (IQR)			
0-Pad	7 (2–52)w	2(1–39)w	< 0.001
0–1 safety Pad	6.5(2–45)w	2(1–29)w	< 0.001

*L-RARP* Transvesical approach for robotic-assisted radical prostatectomy via a bladder neck and prostate combined longitudinal incision, *S-RARP* Standard robot-assisted radical prostatectomy, *IQR* Interquartile range

Tables 5, 6 and 7 displayed EPIC-CP overall, urinary incontinence, and sexual function scores over the study period. L-RARP had significantly improved total EPIC-CP scores at 6 week and 3, 6, 12 and 18 months (*p* < 0.001; Table 5). The EPIC-CP urinary incontinence scores were better for L-RARP at 6 weeks and 3, 6, 12, and 18 months (*p* < 0.001; Table 6). Furthermore, L-RARP had significantly better EPIC-CP sexual function scores (*p* < 0.001; Table 7). Tables 8 and 9 compare baseline versus 18-mo EPIC-CP scores. In patients who received L-RARP, total EPIC-CP and urinary incontinence scores returned to baseline, although EPIC-CP sexual scores did not (3.75 vs. 3.19, *p* = 0.001). In contrast, patients treated with S-RARP had significant differences between baseline and 18-mo urinary incontinence (1.21 vs. 3.42, *p* < 0.001), sexual function (2.99 vs. 4.94, *p* < 0.001), and total EPIC-CP scores (8.13 vs 10.67, *p* < 0.001).

**Table 5:**
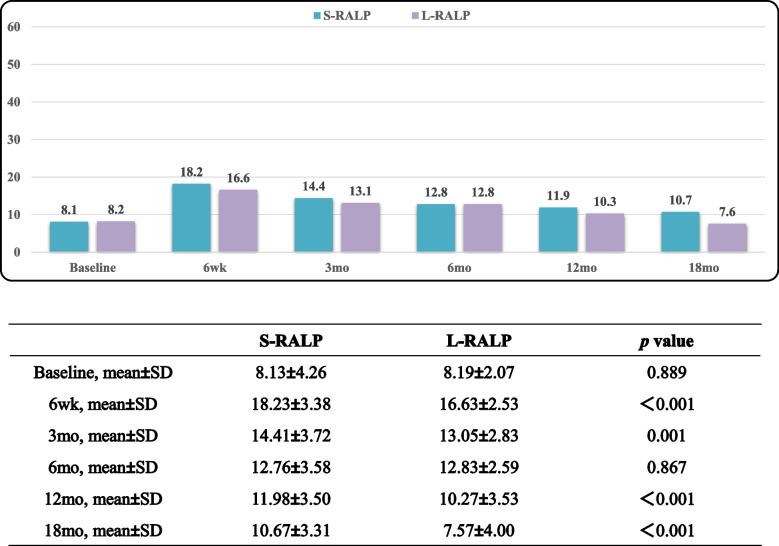
EPIC-CP total QOL scores

**Table 6:**
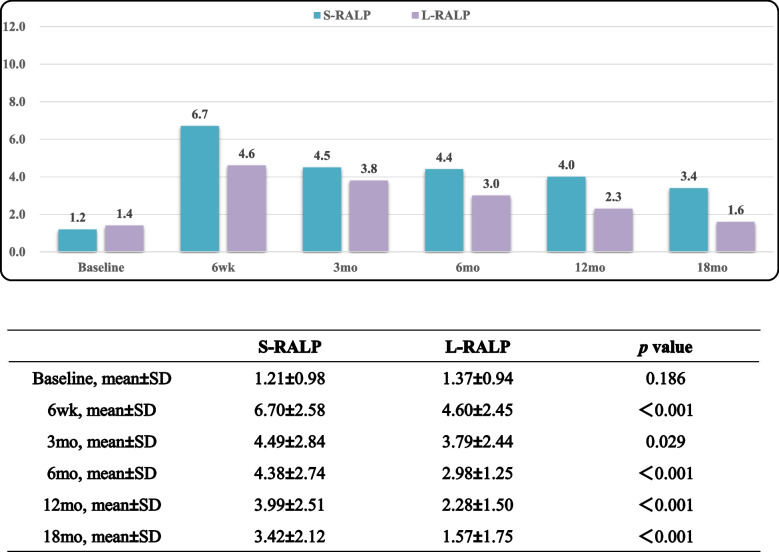
EPIC-CP urinary incontinence scores

**Table 7:**
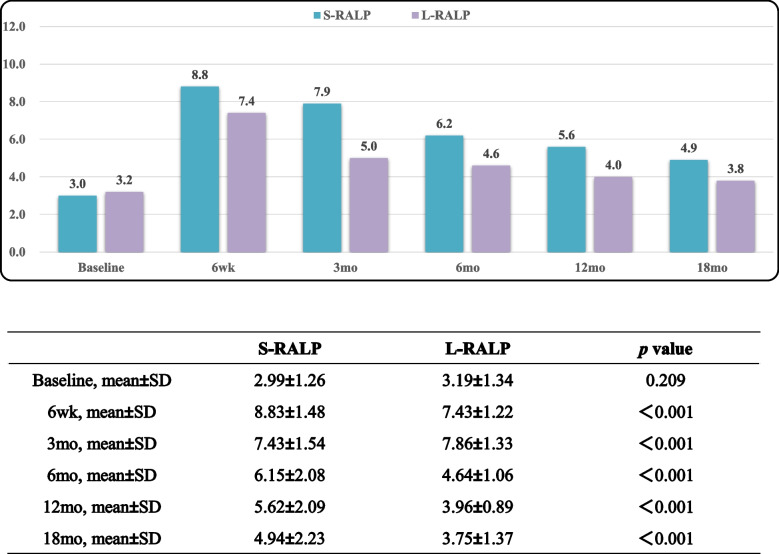
EPIC-CP sexual function scores

**Table 8:**
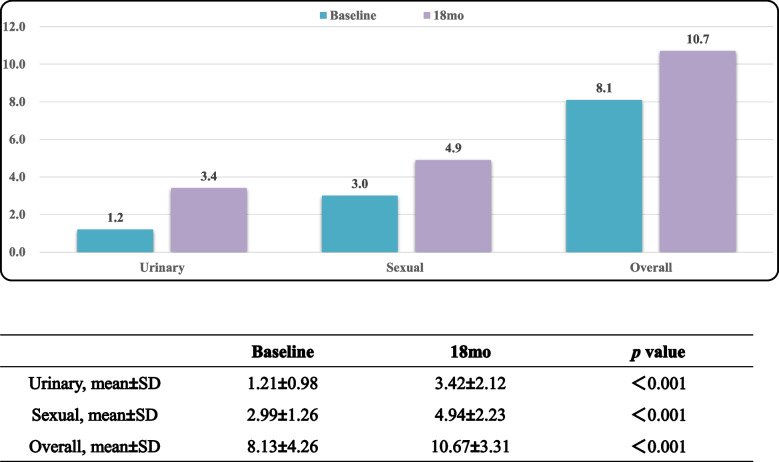
Baseline versus 18-mo EPIC-CP scores in S-RALP group

**Table 9:**
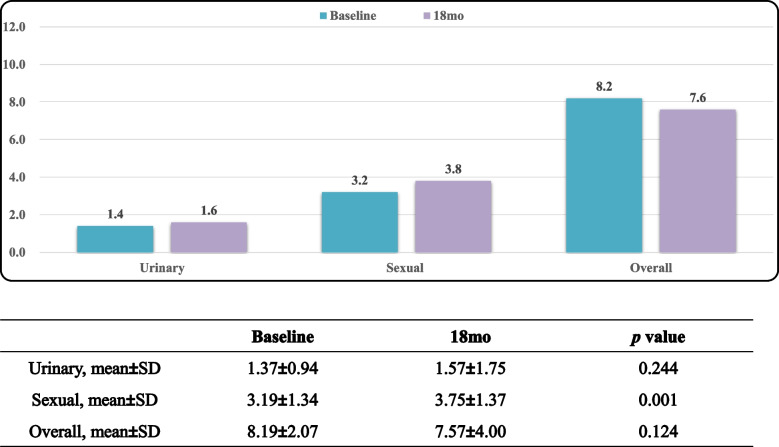
Baseline versus 18-mo EPIC-CP scores in L-RALP group

## Discussion

The traditional robotic laparoscopic prostatectomy is based on the anatomy of the traditional pubic bone posterior approach [6], which may cause damage to the anatomical structures around the prostate, including blood vessels and nerve bundles, the urethra and surrounding sphincter muscles, the puboprostatic ligament, and the prostate’s vascular and venous plexus. This could lead to postoperative sexual dysfunction and urinary incontinence [19–24]. The RALP technique proposed by Bocciardi et al., which preserves the Retzius space, not only has a high degree of accuracy in prostatectomy, effectively reducing the risk of tumor recurrence, but also avoids damage to the above anatomical structures during the surgery, resulting in well surgical results. This new surgical technique is named RALP with Retzius space preservation [15, 25]. To date, there have been plentiful studies comparing the outcomes of Retzius-sparing robotic-assisted radical prostatectomy with standard robotic-assisted radical prostatectomy, which have demonstrated improved continence outcomes for Retzius sparing robotic assisted radical prostatectomy within the first year and equivalent oncologic efficacy out to 18 months. “In addition, neither group of patients in the study had large prostates, which means that the procedure has only been proven for patients with small prostates, studies on large-volume prostate patients need to be compared between S-RALP and L-RALP in the future”.

There are several major technical difficulties in Retzius sparing RALP technique in clinical practice [26–29]: (1) Narrow surgical space: the larger the prostate volume, the narrower the surgical space. (2) This operation requires surgeons with high technical levels. (3) Excessive intraoperative bleeding: Due to the narrow operating space, intraoperative bleeding control is difficult. However, due to the popularity of robotic surgery, S-RALP has gradually improved dramatically in the last decade years indeed, early urinary continence can even reach 87.5% which exchanged for a higher BCR rate as price [30], therefore, it is urgent to find a surgical method with smoother learning curve, lower BCR rate and better urinary control function retention.

In recent years, a new surgical approach has emerged through the improvement of prostate cancer surgery. This approach involves a fascia-sparing radical prostatectomy performed through a combined bladder-prostate longitudinal incision (named, L-RALP), which preserves important urinary control-related tissue structures in the Retzius space. In order to verify the feasibility of the surgery, this study identified 277 patients with histologically-confirmed prostate cancer who underwent either S-RALP or L-RALP and conducted a comparative analysis of the surgical effects between two groups, with a focus on comparing the postoperative sexual and urinary control functions. Based on the postoperative follow-up results and the EPIC-CP scores, we observed the obvious advantages of the L-RALP [31]. First, since L-RALP is a new surgical approach, S-RARP patients underwent surgery later in the S-RARP learning curve compared to L-RARP patients, who underwent surgery early in the L-RARP learning curve. Compared with the S-RALP group, it has a greater advantage in urinary control, because when we define good urinary continence as 0 pads, the urinary continence rate of L-RALP is significantly higher than that of the S-RALP group, even if urinary incontinence was defined as 0–1 safety pad. In addition, the urinary continence function of the L-RALP group showed a trend of improvement in the following 18 months. Moreover, the EPIC-QOL baseline data of the L-RALP group was almost the same as the data at the 18th month, indicating that patients could benefit from L-RALP, making the patient feel that there is no significant change in their quality of life after the surgery.

The intraoperative blood loss in the L-RARP group was more than that in the S-RARP group because the two surgeons used scissors to separate the two sides of the prostate slowly and the venous plexus in the suspensory ligament of the prostate was not sutured, so the blood loss was more than the latter. However, the amount of bleeding is generally controllable, and there is no difference in the blood transfusion rate. The amount of bleeding in the former will further decrease with the increase in operating proficiency. Two surgeons found L-RALP has the following advantages when they performed L-RALP: (1) It does not require the liberation of the bladder and the peri vesical space, and the operation is limited to the deep pelvic space around the prostate. The prostate could be completely resected within the fascia, and the integrity of the vascular and nerve bundles can be fully preserved, which could reduce the damage of radical prostatectomy as much as possible; (2) Intraoperative bleeding is reduced by suture ligation, avoiding the influence of thermal injury on long-term sexual function and urinary continence function; (3) The integrity of the puboprostatic ligament and pudendal artery is preserved; (4) A longitudinal incision is used to open the bladder to more easily expose the vas deferens and seminal vesicles, the separation steps of the bladder neck are reduced, and the damage to the detrusor muscle group is minimized; (5) The bladder neck is easy to identify and retain during the operation, which reduces the incidence of bladder neck contracture and ureteral orifice injury, shortening the indwelling time of the postoperative catheter [18, 32, 33]; (6) The anatomy of the L-RALP starts from the 6 o’clock position of the prostate because the Denonvillier fascia was thicker here, and it is easy to separate with scissors, which can completely preserve the outer fascia and NVB on both sides of the prostate; (7) A sub umbilical incision and 0° mirror can be used for the whole operation. At an extreme angle, a 30° mirror can be considered, or the observation hole mechanical arm raised to improve the field of vision.

According to our experience, there were several points that we need to be cautious during the operation: (1) Preserving the structures around the Retzius gap during the operation: do not open the pelvic fascia or suture the dorsal penile vascular complex, which helps to protect the external urethral sphincter and nerves at the apex of the prostate. (2) Complete interfacial resection of the prostate without opening Disse’s fascia: preserve the prostatic ligament and periprostatic fascia as much as possible to protect the blood vessels and nerves to the greatest extent. When separating the prostate during the operation, try to use a cold knife to slowly separate the prostate to avoid cutting too much tissue in the posterolateral area of the prostate. (3) Accurate dissection of the bladder neck: the boundary between the prostate and the bladder neck should be determined by pulling the catheter and other means, and the neck should be dissected as accurately as possible to reduce damage to the internal urethral sphincter. (4) Double-needle anastomosis of the bladder neck of the urethra: 3–0 double-needle barbed sutures were used for bladder-urethra anastomosis from 6:00 at the lithotomy position to both sides until they meet at 12:00. Because of the membrane sewed into the cause of poor anastomosis. (5) Suture without dead space on the posterior wall of the urethra: when anastomosing the posterior wall of the urethra and bladder, the tissue in the space between the prostate and rectum is brought. On the one hand, it can control the bleeding of the posterior wall; on the other hand, it can strengthen the posterior wall and reduce the occurrence of urinary fistula. (6) Suture the bladder detrusor skirt and puboprostatic ligament tissue on the anterior wall of the urethra: when anastomosing the urethra and the anterior bladder wall, pay attention to suturing the bladder muscle layer and the puboprostatic ligament tissue. This method can strengthen the anterior wall and restore the suspension structure. At the same time, functional reset and morphological reset are achieved.

This surgical method was an improved surgical version of robot-assisted laparoscopic radical prostatectomy under the anterior approach. Although clinicians have verified this modified surgical version, there are still certain difficulties and risks in daily operation. Therefore, highly specialized doctors are required to perform the surgery, and the surgeon needs proper training to ensure the safety and effectiveness of the surgery. However, the surgical operation requires high difficulty. Clinicians can still master it well after going through the learning curves. Although the robot-assisted radical prostatectomy (L-RALP) in the sub fascia through the combined longitudinal incision of the bladder and prostate is theoretically feasible, only patients with localized prostate cancer are suitable for this surgery. Patients who break through the prostate capsule are not suitable for this surgical approach. In addition, the sample size of this study is small, and it is necessary to implement large-sample surgical experimental verification in later tissues.

In conclusion, both S-RALP and L-RALP were safe and effective with similar long-term clinical outcomes in patients with localized prostate cancer. Patients who received L-RALP had significantly better postoperative outcomes including urinary control, and recovery period, suggesting that L-RALP would be an alternative technique for robotic radical prostatectomy.

### Supplementary Information

**Supplementary Material 1. **

## Data Availability

All data generated or analyzed during this study are included in this published article and its supplementary information files.
